# Development of a predictive model to identify patients most likely to benefit from surgery in metastatic breast cancer

**DOI:** 10.1038/s41598-023-30793-8

**Published:** 2023-03-08

**Authors:** Jinfeng Bai, Zeying Li, Junlong Guo, Fuxin Gao, Hui Zhou, Weijie Zhao, Xiang Ma

**Affiliations:** 1 The third affiliated hospital of Kunming Medical University, Kunming, 650118 China; 2 Kunming Medical University, Kunming, China

**Keywords:** Breast cancer, Cancer therapy

## Abstract

Primary tumor resection for metastatic breast cancer (MBC) has demonstrated a survival advantage, however, not all patients with MBC benefit from surgery. The purpose of this study was to develop a predictive model to select patients with MBC who are most likely to benefit from surgery at the primary site. Data from patients with MBC were obtained from the Surveillance, Epidemiology and End Results (SEER) cohort and patients treated at the Yunnan Cancer Hospital. The patients from the SEER database were divided into surgery and non-surgery groups and a 1:1 propensity score matching (PSM) was used to balance baseline characteristics. We hypothesized that patients who underwent local resection of primary tumors had improved overall survival (OS) compared to those who did not undergo surgery. Based on the median OS time of the non-surgery group, patients from the surgery group were further categorized into beneficial and non-beneficial groups. Logistic regression analysis was performed to identify independent factors associated with improved survival in the surgery group and a nomogram was established using the most significant predictive factors. Finally, internal and external validation of the prognostic nomogram was also evaluated by concordance index (C-index) and using a calibration curve. A total of 7759 eligible patients with MBC were identified in the SEER cohort and 92 with MBC patients who underwent surgery at the Yunnan Cancer Hospital. Amongst the SEER cohort, 3199 (41.23%) patients received surgery of the primary tumor. After PSM, the OS between the surgery and non-surgery group was significantly different based on Kaplan–Meier survival analysis (46 vs. 31 months, P < 0.001), In the surgery group, 562 (55.20%) patients survived for longer than 31 months and were classified in the beneficial group. Significant differences were observed in patient characteristics between the beneficial and non-beneficial groups including age, grade, tumor size, liver metastasis, breast cancer subtype and marital status. These factors were used as independent predictors to create a nomogram. The internally and externally validated C-indices of the nomogram were 0.703 and 0.733, respectively, indicating strong consistency between the actual and predicted survival. A nomogram was developed and used to identify MBC patients who are most likely to benefit from primary tumor resection. This predictive model has the potential to improve clinical decision-making and should be considered routine clinical practice.

## Introduction

Breast cancer (BC) is one of the most common tumors in females and the second leading cause of cancer-related deaths in women^1,2^. Approximately 5–8% of patients with BC have distant metastasis at initial presentation^3^. Metastatic breast cancer (MBC) is regarded as an incurable disease and has a 5-year survival of 27%^3,4^. Currently, there are curative outcomes for patients with stage IV BC remain poor yet surgery, chemotherapy, radiotherapy, immunotherapy and targeted therapies are commonly used.

Generally, surgical resection of the primary tumor site is used as a palliative approach in patients with advanced disease, metastatic disease. In stage IV disease, local resection for the primary tumor with or without radiotherapy is not usually taken as a definitive form of treatment. Increasing evidence suggests that locoregional surgery is associated with prolonged survival in some stage IV cancers including the colon, kidney and lung^5–7^. In MBC, several meta-analyses of retrospective studies have shown the potential utility of surgical resection in improving overall survival^8,9^. However, surgical resection is likely to only perform well in MBC patients who are young, in good physical condition and have oligometastatic disease^10^. Also, not all MBC patients may benefit from surgery and the most appropriate method to identify patients most likely to benefit from surgery remains unclear. Currently, the National Clinical Cancer Network (NCCN) guidelines encourage clinicians to perform individualized decisions for local therapy in stage IV disease^11^.

Nomograms have become established as reliable prediction tools that integrate various clinicopathological data to predict the prognosis^12–14^. If the survival result index is replaced with other parameters, the nomogram may be able to predict the outcome of other parameters. In this way, predicting the probability of global parameters obtained by the prognostic nomogram could be used to inform treatment decisions in the clinic. Huang et al.^15^ established a nomogram to predict the probability of bone metastasis in BC patients. Also, a recent study developed a predictive model to screen for the patients most likely to benefit from surgery in advanced lung cancer^16^. However, the optimum methods to select patients with MBC who are most likely to benefit from surgery remain under investigation.

In this study, we aimed to develop a predictive model using data from the Surveillance, Epidemiology and End Results (SEER) database to identify the optimal candidates for primary tumor resection in MBC patients.

## Materials and methods

### Data source and study design

Data were extracted from the Surveillance, Epidemiology and End Results (SEER) database. SEER is a population-based cancer dataset that stores a series of patient and tumor-related data including incidence, survival, mortality and other characteristics^17^. The SEER*Stat software was used to select patients with MBC from 2010 to 2015 based on the site code classifications. MBC patients diagnosed with stage IV disease based on the American Joint Committee on Cancer (AJCC) TNM stage were enrolled in the study who had basic information including active follow-up, known cancer subtype and surgery of the primary tumor. The clinical data for the MBC patients initially diagnosed at Yunnan Cancer Hospital between January 2012 and August 2016 were retrospectively collected and analyzed. All methods were performed in accordance with the relevant guidelines and regulations. The research was approved by the ethics committee of Yunnan Cancer Hospital (ethical clearance No: KYLX202123, which also waived the need for informed consent due to retrospective nature of the study).

The exclusion criteria for the study were (1) age < 18 years, (2) unknown follow-up times, (3) unknown differentiation grade, (4) undefined TNM staging, (5) unknown histology, (6) unknown tumor size, (7) unknown marital status, (8) unknown race,; (9) unknown survival duration and (10) patients with previous cancers or other tumors.

### Statistical analyses

Our study samples were classified into surgery and non-surgery groups depending on whether they had received surgery of the primary tumor. The variables that could potentially influence treatment responses were extracted including demographics (age, race and gender), the year of diagnosis, marital status, socioeconomic status (SES), primary tumor site, tumor grade, tumor size, TNM stage, BC subtype, surgery of the primary tumor, chemotherapy, radiation therapy, distant metastatic site (bone, brain, liver, lung and multiple organs) and survival (time and status). These parameters were used to create a composite SES variable similar to previous studies^18–20^ that included education, family income and poverty level. Propensity score matching (PSM) was used to reduce selection bias between the treatment groups and allow direct comparison of the data. The patients in both groups were 1:1 matched with a caliper of 0.001. Categorical variables were evaluated using a chi-squared test or a Fisher’s exact test.

Overall survival (OS) was calculated from the time of diagnosis to the time of death. OS between the surgery and the non-surgery groups were evaluated from the Kaplan–Meier curves and the data were compared using a log-rank test. Univariate and multivariate cox proportional hazards regression was used to identify the independent risk factors. The parameters referred to hazard ratios (HRs) with 95% confidence intervals (CIs). Statistical analyses were conducted using SPSS software (version 26.0) and a P-value threshold of < 0.05 was regarded as statistically significant.

### Development and validation of the predictive nomogram

We hypothesized that patients who underwent local resection of primary tumors has longer OS compared to those who did not receive surgery. Based on the median OS time (31 months) of patients in the non-surgery group after PSM, the eligible patients who had received surgery of primary tumors were categorized into beneficial and non-beneficial groups. Using multivariate analysis, we screened for independent risk factors that may improve the survival time (31 months) and evaluated these factors before surgery. These factors included age, grade, tumor size, liver metastasis, BC subtype and marital status. Based on the results of the multivariate logistic analysis, a nomogram was constructed to predict MBC candidates most likely to benefit from surgery.

The predictive capability of the nomogram was evaluated using the concordance index (C-index) where larger values indicate more accurate prediction. An external validation cohort of independent Chinese patients was used to further access the discrimination and calibration of the nomogram. The concordance between the predicted and the actual probabilities were internally and externally measured using calibration curves and 1000 bootstrap resamples. These analyses were performed using R software (version 4.0.5, http://www.Rproject.org) with the “rms” package.

Based on the results of the predictive model, all MBC patients in the SEER database after PSM were assigned to three groups; a beneficial surgery group with > 50% of patients assuming probability of benefit, a non-beneficial surgery group with < 50% of patients assuming the probability of benefit, and a non-surgery group. Kaplan–Meier analyses were performed to test the ability of the predictive model to distinguish patients who could benefit from surgical resection of the primary tumor.

### Ethics approval and consent to participate

The research was approved by the ethics committee of Yunnan Cancer Hospital (ethical clearance No: KYLX202123, which also waived the need for informed consent due to retrospective nature of the study).

## Results

### Patient characteristics

**Figure 1:**
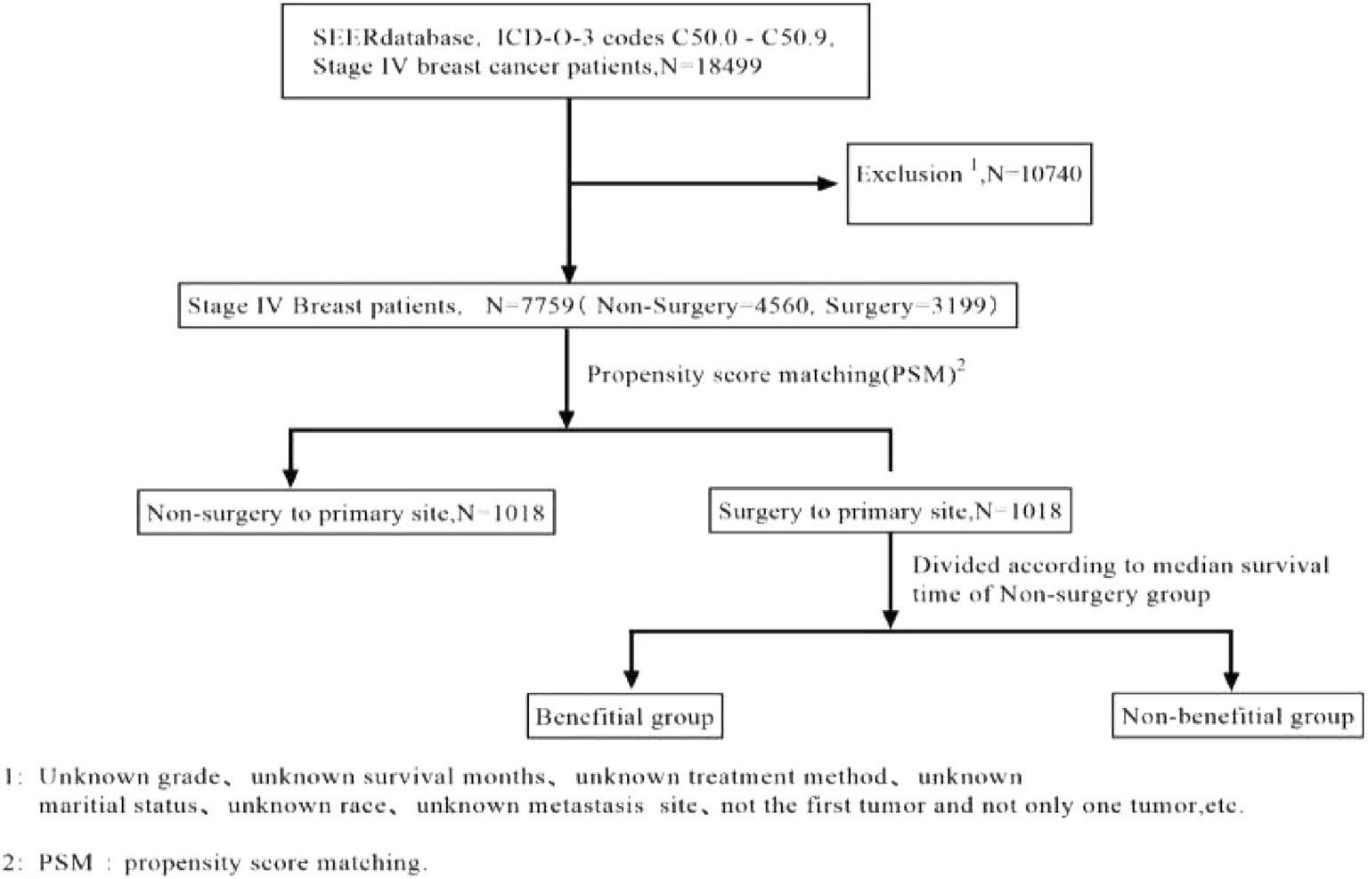
A flow diagram showing the study selection process.

The data from a total of 18,499 MBC patients were collected from the SEER database. An additional 92 patients with MBC who underwent surgery at the Yunnan Cancer Hospital were also included in this study. From the SEER datasets, a rigorous filtering procedure was used and ultimately 7759 eligible patients were identified and included in the study (Fig. 1). As shown in Table 1, the patients were categorized into surgery (n = 3199) and non-surgery groups (n = 4560) based on surgery to the primary tumor. There were differences in the age, year of diagnosis, socioeconomic status, grade, tumor size, T Stage, N Stage, chemotherapy, radiotherapy, metastasis pattern, breast subtype and marital status between both groups before PSM. Surgery was mainly associated with younger patients and those with lower TNM stage indicating an imbalance in the baseline characteristics between surgery and non-surgery groups.

**Table 1:** The patient demographics and clinic-pathologic characteristics before and after PSM.

Demographic or characteristic	Before PSM				After PSM			
	Total	Surgery	Non-surgery	P-value	Total	Surgery	Non-surgery	P-value
	n = 7759	n = 3199 (N%)	n = 4560 (N%)		n = 2036	n = 1018 (N%)	n = 1018 (N%)	
Age								
< 60	3962	1777 (55.5%)	2185 (47.9%)	< 0.001	1067	533 (52.4%)	534 (52.5%)	0.965
≥ 60	3797	1422 (44.5%)	2375 (52.1%)		969	485 (47.6%)	484 (47.5%)	
Race								
White	5756	2377 (74.3%)	3379 (74.1%)	0.229	1519	763 (75%)	756 (74.3%)	0.357
Black	1349	536 (16.8%)	813 (17.8%)		321	166 (16.3%)	155 (15.2%)	
Other^1^	654	286 (8.9%)	368 (8.1%)		196	89 (8.7%)	107 (10.5%)	
Sex								
Male	93	43 (1.3%)	50 (1.1%)	0.324	22	14 (1.4%)	8 (0.8%)	0.198
Female	7666	3156 (98.7%)	4510 (98.9%)		2014	1004 (98.6%)	1010 (99.2%)	
Year of diagnosis								
2010–2012	3594	1716 (53.6%)	1878 (41.2%)	< 0.001	993	498 (48.9%)	495 (48.6%)	0.894
2013–2015	4165	1483 (46.4%)	2682 (58.8%)		1043	520 (51.1%)	523 (51.4%)	
Socioeconomic status								
High SES	3136	1212 (37.9%)	1924 (42.2%)	< 0.001	735	362 (35.6%)	373 (63.6%)	0.612
Low SES	4623	1987 (62.1%)	2636 (57.8%)		1301	656 (64.4%)	645 (63.4%)	
Grade								
Grade I	549	182 (5.7%)	367 (8.0%)	< 0.001	85	45 (4.4%)	40 (3.9%)	0.684
Grade II	3135	1084 (33.9%)	2051 (45.0%)		875	429 (42.1%)	446 (43.8%)	
Grade III	4025	1911 (59.7%)	2114 (46.4%)		1076	544 (53.4%)	532 (52.3%)	
Grade IV	50	22 (0.7%)	28 (0.6%)		0	0 (0%)	0 (0%)	
Tumor size (mm)								
< 20	937	354 (11.1%)	583 (12.8%)	0.047	180	94 (9.2%)	86 (8.4%)	0.100
20–50	3880	1637 (51.2%)	2243 (49.2%)		1128	540 (53.0%)	588 (57.8%)	
> 50	2942	1208 (37.8%)	1734 (38%)		728	384 (37.7%)	344 (33.8%)	
T stage								
T_1_	948	368 (11.5%)	580 (12.7%)	< 0.001	188	96 (9.4%)	92 (9.0%)	0.821
T_2_	2879	1300 (40.6%)	1579 (34.6%)		876	428 (42.0%)	448 (44.0%)	
T_3_	1498	616 (19.3%)	882 (19.3%)		310	160 (15.7%)	150 (14.7%)	
T_4_	2434	915 (28.6%)	1519 (33.3%)		662	334 (32.8%)	328 (32.2%)	
N stage								
N_0_	1655	520 (16.3%)	1135 (24.9%)	< 0.001	401	203 (19.9%)	198 (19.4%)	0.681
N_1_	3649	1218 (38.1%)	2431 (53.3%)		1050	521 (51.2%)	529 (52.0%)	
N_2_	1064	653 (20.4%)	411 (9.0%)		231	123 (12.1%)	108 (10.6%)	
N_3_	1391	808 (25.3%)	583 (12.8%)		354	171 (16.8%)	183 (18.0%)	
Chemotherapy								
Yes	2938	931 (29.1%)	2007 (44.0%)	< 0.001	778	395 (38.8%)	383 (37.6%)	0.584
No/unknown	4821	2268 (70.9%)	2553 (56.0%)		1258	623 (61.2%)	635 (62.4%)	
Radiotherapy								
Yes	4967	1711 (53.5%)	3256 (71.4%)	< 0.001	1391	694 (68.2%)	697 (68.5%)	0.886
No/unknown	2792	1488 (46.5%)	1304 (28.6%)		645	324 (31.8%)	321 (31.5%)	
Metastasis pattern (yes vs. no)								
Liver metastasis	1965	633 (19.8%)	1332 (29.2%)	< 0.001	335	161 (15.8%)	174 (17.1%)	0.437
Brain metastasis	473	113 (3.5%)	360 (7.9%)	< 0.001	35	17 (1.7%)	18 (1.8%)	0.865
Bone metastasis	4947	1805 (56.4%)	3142 (68.9%)	< 0.001	1336	670 (65.8%)	666 (65.4%)	0.852
Lung metastasis	2367	791 (24.7%)	1576 (34.6%)	< 0.001	505	237 (23.3%)	268 (26.3%)	0.112
Multiple sites	2295	579 (18.1%)	1716 (37.6%)	< 0.001	353	174 (17.1%)	179 (17.6%)	0.770
Breast subtype								
HR + /HER2-	4445	1730 (54.0%)	2720 (59.5%)	< 0.001	1329	661 (64.9%)	668 (65.6%)	0.727
HR + /HER2 +	1383	569 (17.8%)	819 (17.9%)		295	142 (13.9%)	153 (15.0%)	
HR-/HER2 +	739	325 (10.1%)	411 (9.0%)		154	79 (7.8%)	75 (7.4%)	
HR-/HER2-	1192	581 (18.1%)	618 (13.5%)		258	136 (13.4%)	122 (12.0%)	
Location								
Central^2^	562	233 (7.3%)	329 (7.2%)	0.536	132	74 (7.3%)	58 (5.7%)	0.638
Upper	2684	1111 (34.7%)	1573 (34.5%)		687	340 (33.4%)	347 (34.1%)	
Lower	823	353 (11.0%)	470 (10.3%)		231	115 (11.3%)	116 (11.4%)	
Axillary tail	48	22 (0.7%)	26 (0.6%)		11	5 (0.5%)	6 (0.6%)	
Overlapping	1750	733 (22.9%)	1017 (22.3%)		476	245 (24.1%)	231 (22.7%)	
Other^3^	1892	747 (23.4%)	1145 (25.1%)		499	239 (23.5%)	260 (25.5%)	
Marital status								
Unmarried	4057	1528 (47.8%)	2529 (55.5%)	< 0.001	1065	536 (52.7%)	529 (52.0%)	0.756
Married	3702	1671 (52.2%)	2031 (44.5%)		971	482 (47.3%)	489 (48.0%)	

*1: American Indian/AK Native, Asian/Pacific Islander; 2: central portion of breast or nipple; 3: Breast,NOS.

A 1:1 PSM was performed to rematch the datasets. 1018 MBC patients treated with surgery of the primary site and 1018 MBC patients who did not undergo surgery were enrolled in the study following the 1:1 PSM analysis and the baseline characteristics were all balanced (all *P* > 0.05). Most of these patients were white with grade III tumors. The upper part of the breast was the most frequent location of the primary tumor. The largest proportion of BC subtypes were luminal A (HR + /HER2-) tumors and bone metastasis occurred in the majority of patients. Besides, no noticeable differences was observed between surgery cohort and Chinese cohort (all *P* > 0.05) (Table 2).

**Table 2:** Patient demographics and clinicopathologic characteristics in surgery cohort and Chinese validation cohort.

Demographic or characteristic	Total	Surgery cohort	Chinese cohort	P-value
	n = 1111 (100%)	n = 1018 (100%)	n = 93 (100%)	
Age				
< 60	586 (52.7%)	533 (52.4%)	53 (57.0%)	0.392
≥ 60	525 (47.3%)	485 (47.6%)	40 (43.0%)	
Sex				
Male	14 (1.3%)	14 (1.4%)	0 (0.0%)	0.255
Female	1097 (98.7%)	1004 (98.6%)	93 (100%)	
Year of diagnosis				
2010–2012	538 (48.4%)	498 (48.9%)	40 (43.0%)	0.275
2013–2015	573 (51.6%)	520 (51.1%)	53 (57.0%)	
Socioeconomic status				
High SES	398 (35.8%)	362 (35.6%)	36 (38.7%)	0.544
Low SES	713 (64.2%)	656 (64.4%)	57 (61.3%)	
Grade				
Grade I	48 (4.3%)	45 (4.4%)	3 (3.2%)	0.586
Grade II	473 (42.6%)	429 (42.1%)	44 (47.3%)	
Grade III	590 (53.1%)	544 (53.4%)	46 (49.5%)	
Tumor size (mm)				
< 20	108 (9.7%)	94 (9.2%)	14 (15.1%)	0.187
20–50	585 (52.7%)	540 (53.0%)	45 (48.4%)	
> 50	418(37.6%)	384 (37.7%)	34 (36.6%)	
T stage				
T_1_	108 (9.7%)	96 (9.4%)	12 (12.9%)	0.732
T_2_	466 (41.9%)	428 (42.0%)	38 (40.9%)	
T_3_	175 (15.8%)	160 (15.7%)	15 (16.1%)	
T_4_	362 (32.6%)	334 (32.8%)	28 (30.1%)	
N stage				
N_0_	216 (19.4%)	203 (19.9%)	13 (14.0%)	0.338
N_1_	570 (51.3%)	521 (51.2%)	49 (52.7%)	
N_2_	133 (12.0%)	123 (12.1%)	10 (10.8%)	
N_3_	192 (17.3%)	171 (16.8%)	21 (22.6%)	
Chemotherapy				
Yes	433 (39.0%)	395 (38.8%)	38 (40.9%)	0.697
No/unknown	678 (61.0%)	623 (61.2%)	55 (59.1%)	
Radiotherapy				
Yes	757 (68.1%)	694 (68.2%)	63 (67.7%)	0.932
No/unknown	354 (31.9%)	324 (31.8%)	30 (32.3%)	
Metastasis pattern (yes vs. no)				
Liver metastasis	170 (15.3%)	161 (15.8%)	9 (9.7%)	0.116
Brain metastasis	19 (1.7%)	17 (1.7%)	2 (2.2%)	0.732
Bone metastasis	730 (65.7%)	670 (65.8%)	60 (64.5%)	0.801
Lung metastasis	261 (23.5%)	237 (23.3%)	24 (25.8%)	0.582
Multiple sites	194 (17.5%)	174 (17.1%)	20 (21.5%)	0.283
Breast subtype				
HR + /HER2-	713 (64.2%)	661 (64.9%)	52 (55.9%)	0.127
HR + /HER2 +	160 (14.4%)	142 (13.9%)	18 (19.4%)	
HR-/HER2 +	91 (8.2%)	79 (7.8%)	12 (12.9%)	
HR-/HER2-	147 (13.2%)	136 (13.4%)	11 (11.8%)	
Location				
Central^2^	80 (7.2%)	74 (7.3%)	6 (6.5%)	0.101
Upper	372 (33.5%)	340 (33.4%)	32 (34.4%)	
Lower	125 (11.3%)	115 (11.3%)	10 (10.8%)	
Axillary tail	8 (0.7%)	5 (0.5%)	3 (3.2%)	
Overlapping	265 (23.9%)	245 (24.1%)	20 (21.5%)	
Other^3^	261 (23.5%)	239 (23.5%)	22 (23.7%)	
Marital status				
Unmarried	577 (51.9%)	536 (52.7%)	41 (44.1%)	0.113
Married	534 (48.1%)	482 (47.3%)	52 (55.9%)	

*1: American Indian/AK Native, Asian/Pacific Islander; 2: central portion of breast or nipple; 3: Breast,NOS.

### The efficacy of primary tumor resection on OS

**Figure 2:**
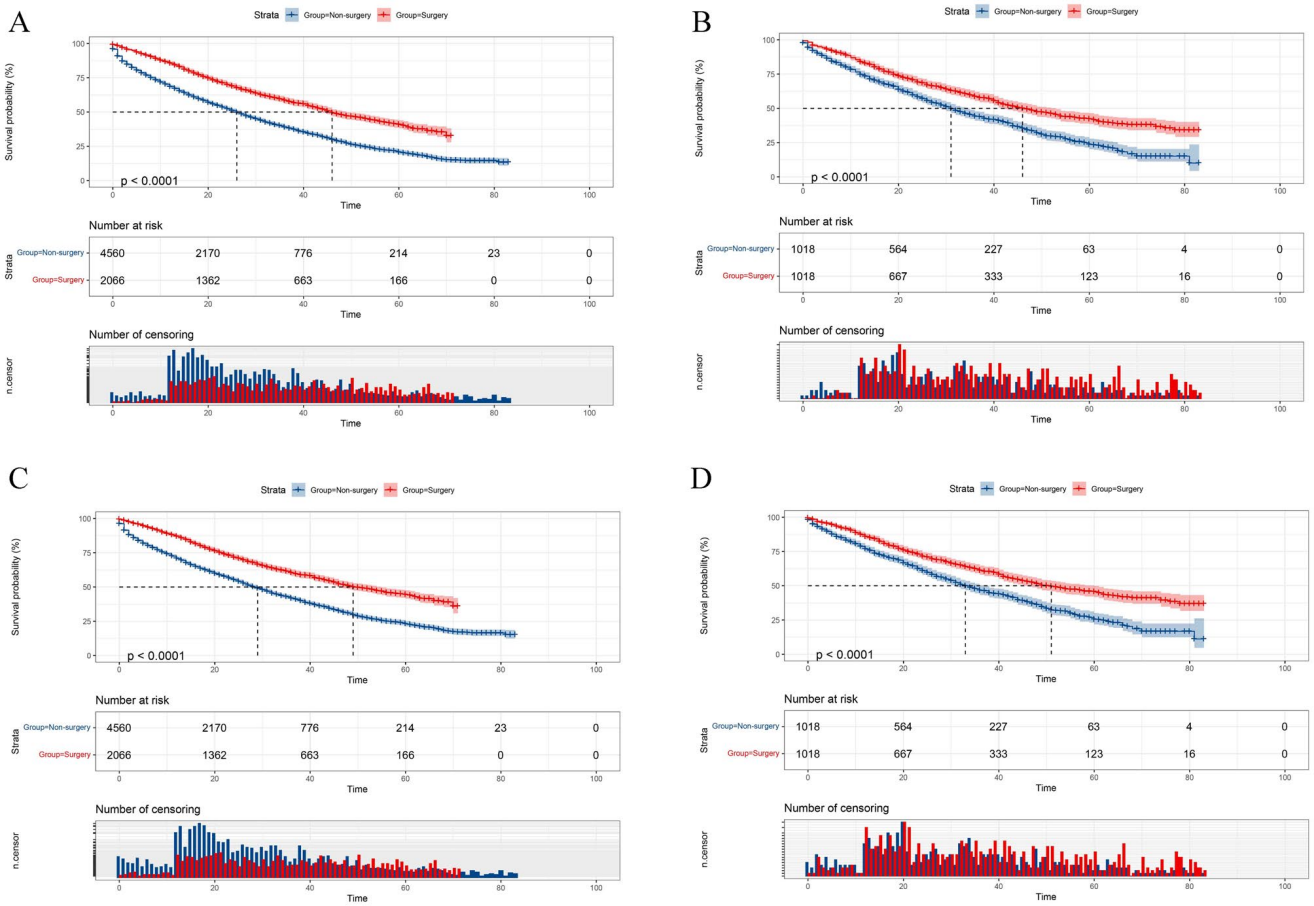
Kaplan–Meier survival analysis of OS in the surgery and non-surgery group before PSM (**A**) and after PSM (**B**), and CSS in the surgery and non-surgery group before PSM (**C**) and after PSM (**D**).

The Kaplan–Meier curves comparing the OS in the surgery and non-surgery groups are shown in Fig. 2A,B. MBC patients who underwent primary tumor resection had significantly higher OS rates than patients who did not receive surgery (*P* < 0.001). After PSM, the median survival time was 46 months (95% CI  41.56–50.44) for patients in the surgery group compared to 31 months (95% CI  28.08–33.92) for patients in the non-surgery group. Besides, cancer-specific survival (CSS) in the two groups was also compared by the Kaplan Meier survival curve, and the result showed a significant difference before PSM and after PSM (Fig. 2C,D).

### Surgery to the primary site as an independent prognostic factors in MBC

Among the patient populations after PSM, the results of the univariate analysis in OS showed that age, race, grade, socioeconomic status, tumor size, T Stage, N Stage, radiotherapy, metastasis pattern, BC subtype, marital status and surgery to the primary tumor were significant prognostic factors in MBC patients. Furthermore, factors with P-values < 0.05 from the univariate analysis were incorporated into the multivariate Cox analysis. The results showed that surgery to the primary tumor was an independent prognostic factor associated with better OS (HR = 0.60, 95% CI 0.54–0.68, *P* < 0.001). Age, grade, N stage, radiotherapy, metastasis pattern, BC subtype and marital status were also confirmed as independent predictive factors for OS in MBC patients (Table 3).

**Table 3:** Cox proportional hazards regression model analysis of OS in the PSM population.

Variables	Univariate analysis			Multivariate analysis		
	HR	95% CI	P	HR	95% CI	P
Age						
< 60	1			1		
≥ 60	1.454	1.289–1.640	< 0.001	1.448	1.278–1.642	< 0.001
Race						
White	1			1		
Black	1.325	1.131–1.552	< 0.001	1.163	0.986–1.373	0.073
Other^1^	0.879	0.707–1.092	0.243	0.915	0.735–1.140	0.430
Sex						
Male	1			-		
Female	0.889	0.503–1.571	0.685			
Grade						
Grade I	1			1		
Grade II	1.328	0.934–1.886	0.114	1.31	0.918–1.869	0.136
Grade III	2.292	1.622–3.238	< 0.001	2.074	1.447–2.972	< 0.001
Socioeconomic status(SES)						
High-ESE	1			1		
Low- ESE	1.15	1.013–1.304	0.03	1.063	0.935–1.210	0.349
Tumor size (mm)						
< 20	1			1		
20–50	1.109	0.877–1.403	0.386	0.952	0.661–1.371	0.790
> 50	1.612	1.271–2.046	< 0.001	1.114	0.763–1.627	0.575
T Stage						
T_1_	1			1		
T_2_	1.108	0.872–1.408	0.401	1.142	0.784–1.662	0.489
T_3_	1.406	1.077–1.834	0.012	1.263	0.834–1.912	0.270
T_4_	1.786	1.406–2.270	< 0.001	1.374	0.942–2.003	0.099
N stage						
N_0_	1			1		
N_1_	0.906	0.772–1.062	0.223	0.810	0.684–0.960	0.015
N_2_	0.961	0.769–1.201	0.726	0.809	0.637–1.028	0.082
N_3_	1.229	1.017–1.484	0.033	0.907	0.734–1.120	0.365
Chemotherapy						
No/unknown	1			–		
Yes	0.900	0.796–1.017	0.090			
Radiotherapy						
No/unknown	1			1		
Yes	0.809	0.709–0.922	0.001	0.858	0.745–0.989	0.034
Metastasis pattern (yes vs. no)						
Liver metastasis	1.357	1.162–1.585	< 0.001	1.369	1.097–1.709	0.005
Brain metastasis	2.101	1.461–3.021	< 0.001	1.872	1.253–2.796	0.002
Bone metastasis	0.824	0.727–0.934	0.003	1.111	0.923–1.337	0.265
Lung metastasis	1.498	1.313–1.709	< 0.001	1.098	0.904–1.333	0.347
Multiple sites	1.550	1.342–1.791	< 0.001	1.237	0.943–1.624	0.125
Breast subtype						
HR + /HER2-(Luminal A)	1			1		
HR + /HER2 + (Luminal B)	0.710	0.583–0.864	0.001	0.524	0.440–0.668	< 0.001
HR-/HER2 + (HER2 enriched)	0.879	0.683–1.132	0.317	0.605	0.458–0.800	< 0.001
HR-/HER2-(Triple negative)	2.633	2.245–3.089	< 0.001	1.900	1.574–2.295	< 0.001
Location						
Central^2^	1			–		
Upper	1.037	0.796–1.351	0.789			
Lower	1.044	0.771–1.414	0.780			
Axillary tail	1.001	0.403–2.487	0.998			
Overlapping	1.102	0.839–1.447	0.487			
Other^3^	1.294	0.988–1.695	0.062			
Marital status						
Unmarried	1			1		
Married	0.687	0.608–0.776	< 0.001	0.763	0.672–0.866	< 0.001
Surgery to primary site						
No	1			1		
Yes	0.633	0.561–0.715	< 0.001	0.604	0.535–0.683	< 0.001

*1: American Indian/AK Native, Asian/Pacific Islander; 2: central portion of breast or nipple; 3: Breast, NOS.

### Factors associated with benefit from surgery in MBC patients

**Figure 3:**
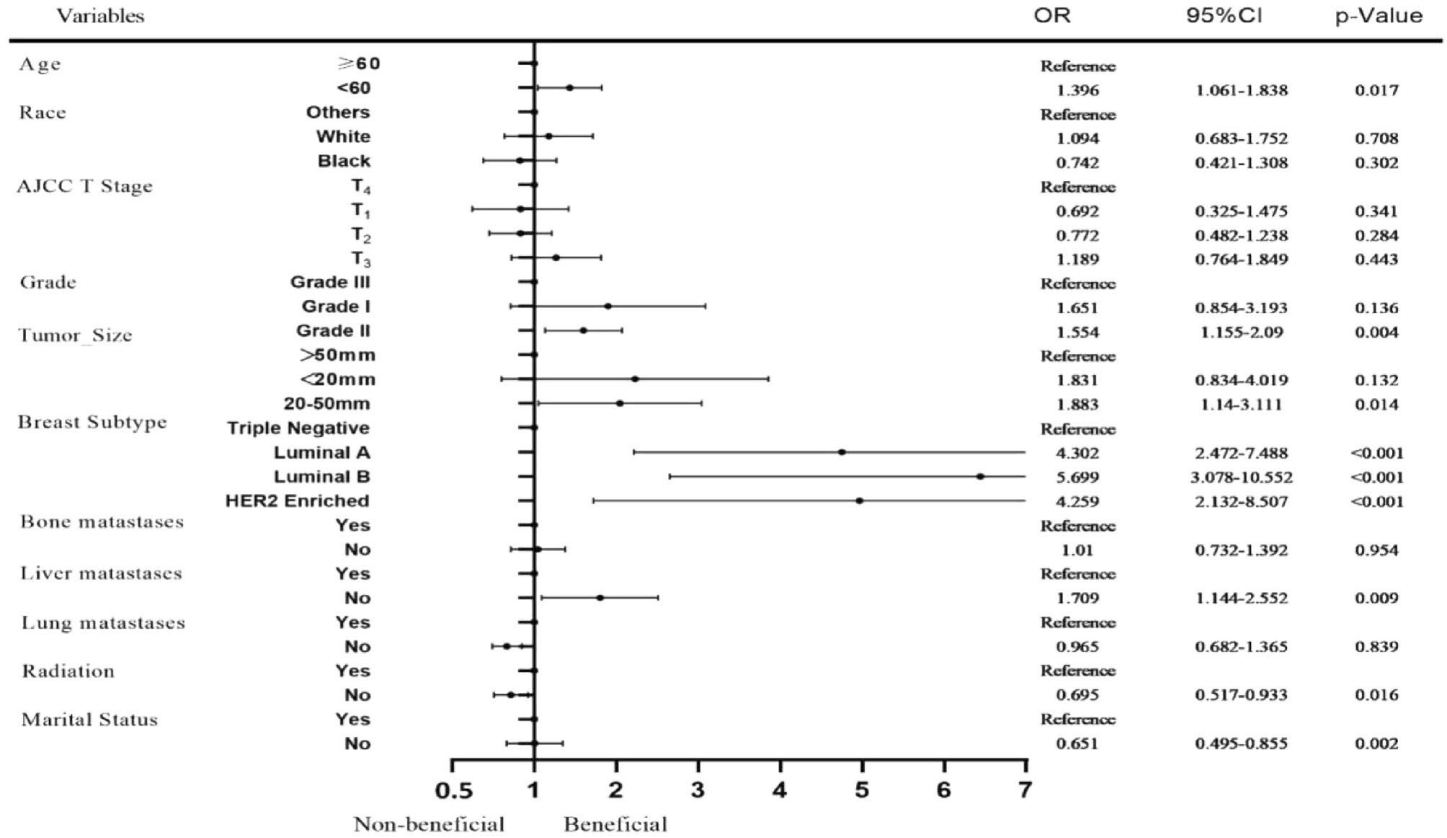
Forest plots of the multivariate logistic regression analysis.

Multivariate logistic regression analysis was used to identify the factors associated with benefit from surgery and the data are presented in Fig. 3. The outcomes showed that age, grade, tumor size, radiotherapy, liver metastasis, BC subtype and marital status were independent predictors for surgical intervention in MBC patients (*P* < 0.05).

### Development of a nomogram to determine optimal candidates for primary tumor resection

Patients who underwent local resection of the primary tumor are assumed to live longer than those who did not surgery. In the surgery cohort, 562 (55.20%) patients lived longer than 31 months who were classified as the beneficial group. Patients who lived < 31 months in the surgery cohort were assigned to the non-beneficial group.

**Figure 4:**
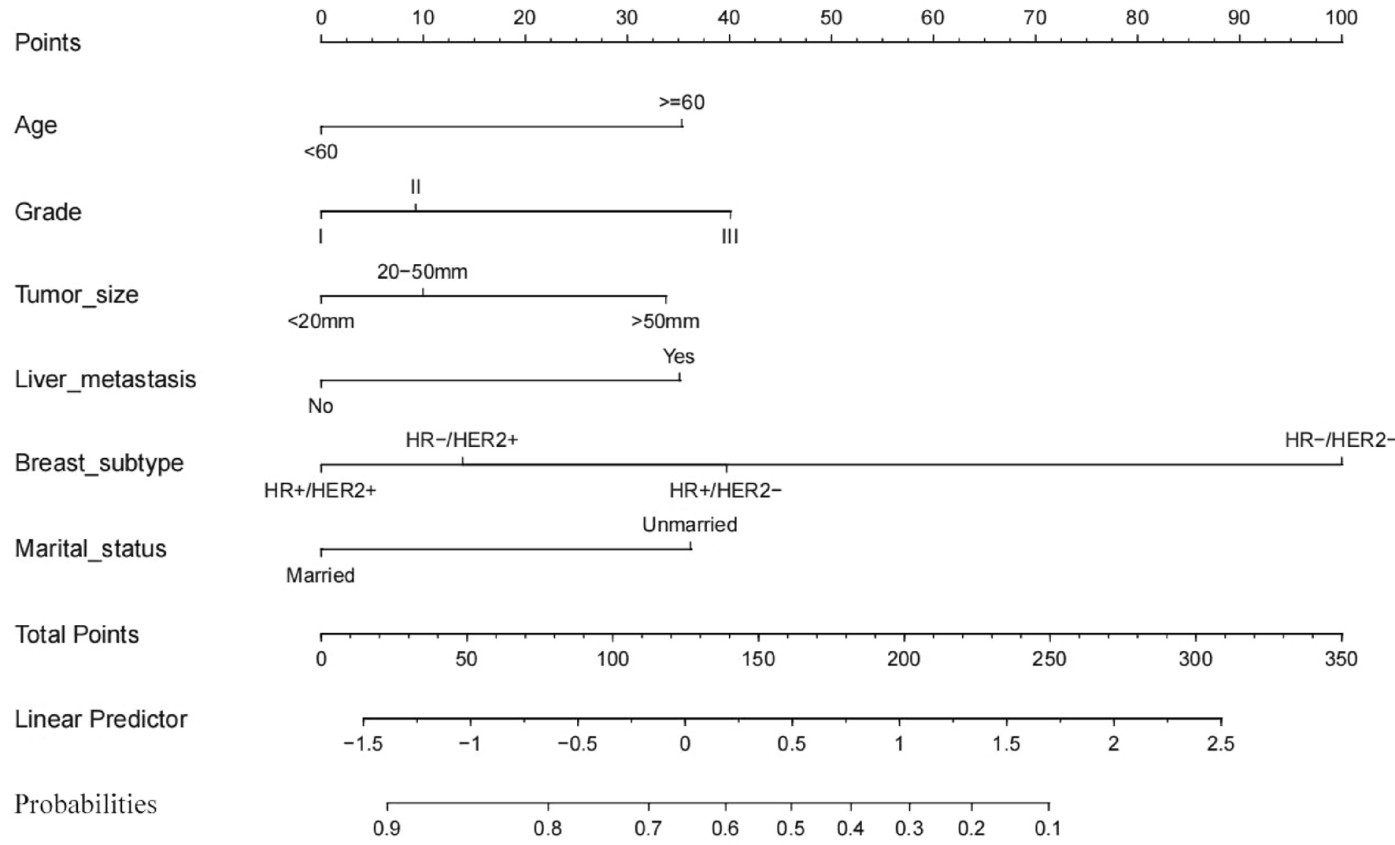
A nomogram to predict optimal candidates for primary tumor resection in MBC patients.

Based on the beneficial and non-beneficial groups, we screened the independent risk factors associated with OS in multivariate logistic regression analysis. This approach was used to assess associations with surgery, including age, grade, tumor size, liver metastasis, BC subtype and marital status. The key independent factors were integrated into a nomogram to predict MBC patients most likely to benefit from primary tumor resection. This model indicated that BC subtype had the largest impact on prognosis, followed by grade and marital status. Other factors including age, tumor size and liver metastasis also had a moderate influence on survival. The specific scoring system of the nomogram is shown in Fig. 4.

### Nomogram validation

**Figure 5:**
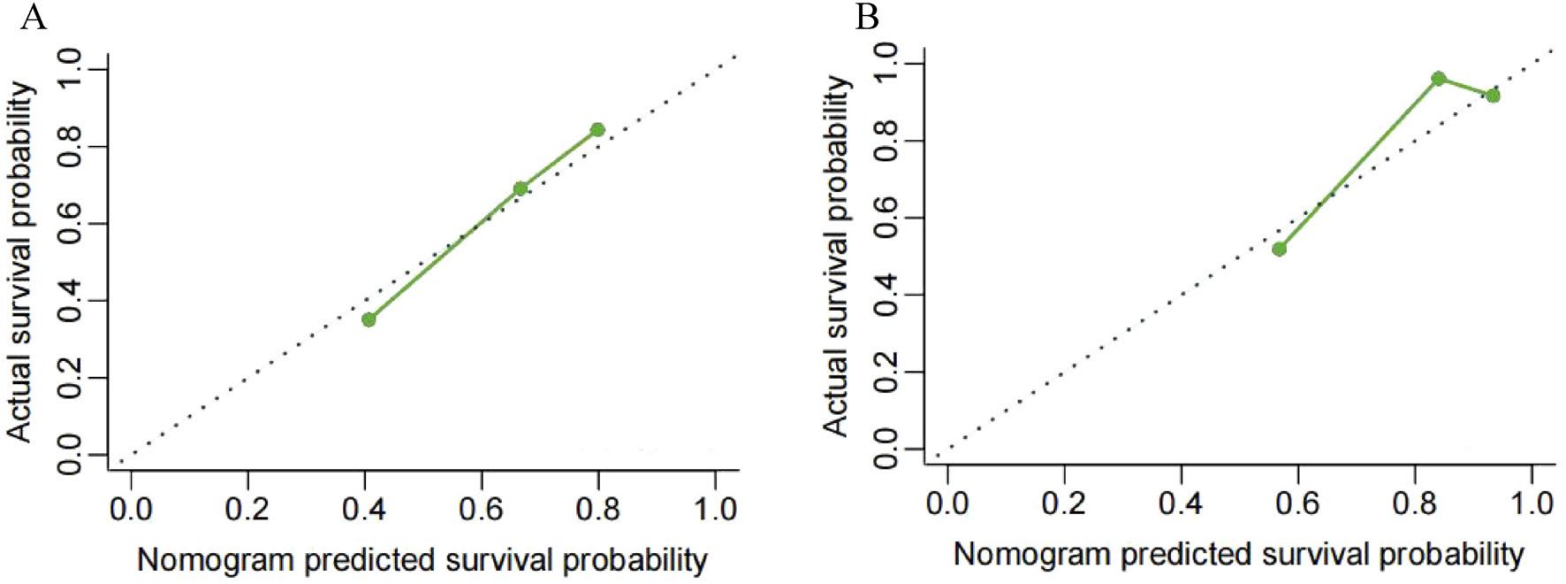
Calibration curves of the nomogram to predict the patients most likely to benefit from surgery in the SEER (**A**) and the Chinese cohorts (**B**).

Internal validation showed that the nomogram could accurately predict OS survival with a C-index of 0.703 (95% CI  0.691–0.716). Similarly, external validation using the independent Chinese cohort showed that the C-index for the nomogram was 0.733 (95% CI  0.690–0.781). The internal and external calibration plots showed optimal consistency between the predicted and observed results (Fig. 5). We further validated the ability of the predictive model in the Chinese patient cohort.

**Figure 6:**
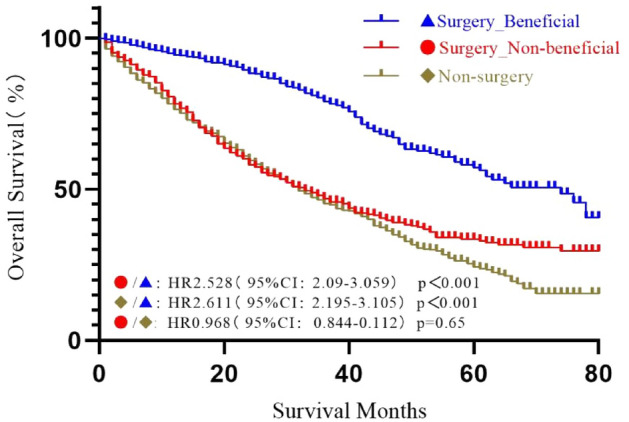
Kaplan–Meier curves to differentiate beneficial and non-beneficial groups based on the predictive model in patients from the SEER database after PSM.

The OS in the SEER three groups after PSM was assessed from the Kaplan–Meier curves and compared using a log-rank test (Fig. 6). The results failed to show significant differences between the non-surgery and non-beneficial surgery groups (*P* > 0.05). However, the patients in the beneficial surgery group had a significantly longer survival time compared to the non-beneficial surgery (HR = 2.528, 95% CI  2.090–3.059, *P* < 0.001) or the non-surgery groups (HR = 2.611, 95% CI  2.195–3.105, *P* < 0.001).

### Clinical practice

The probability of benefit from surgery of the primary site may be obtained by adding the points of each variable. For example, if a patient is a 65-year-old female who married and diagnosed with moderately differentiated BC with liver metastasis that is classified as T2NxM1 stage and the BC subtype is HR + /HER2-, the total score would be calculated as 130 points and the probability that the patient would benefit from surgery is 63%.

## Discussion

The treatment of patients with MBC is highly complex due to heterogeneity of the disease. There is an urgent need for tailored treatment strategies based the individual biology, metastatic burden and social status of pateints. Traditionally, primary tumor resection is used with palliative intent in the treatment of MBC. However, emerging evidence suggests that surgical resection of the primary tumor may improve OS in patients with MBC^21–23^. Consistent with our findings, Zhao et al.^24^ analyzed the data of 7986 patients with de novo MBC from the SEER database between 2010 and 2016 and demonstrated a survival benefit in patients who received surgery compared to those who did not. In their study^24^, nine independent factors, including age, race, marital status, grade, breast subtype, T stage, metastatic site, surgery, and chemotherapy, were found to be independent prognostic indicators of the OS of MBC patients. Of these, their nomogram showed that breast subtype had greatest impact on prognosis while primary site surgery had a moderate impact on prognosis. Similarly, Cui et al.^25^ divided stage IV BC patients into four groups based on the different surgical procedures and reported that the use of surgery was related to a lower probability of breast cancer-specific death in patients with stage IV BC. In addition, the authors also determined the factors associated with bone-only and visceral metastases in BC patients using competing risk models, resulting in the development of two competing risk nomograms. Both these nomograms reflected that, apart from breast subtype, surgery of the primary site appeared to be the most influential factor in assessing the prognosis of stage IV BC patients. However, not all MBC patients appear to benefit equally from surgery and the effects of primary tumor resection should be clarified for those patients. Primary tumor resection is beneficial in reducing tumor burden and metastatic spread^26^ but may contribute to the restoration of immunocompetence as anti-cancer immune responses can be modulated by the primary tumor^27^. So it is necessary to identify which patients are most likely to benefit based on their clinical features.

In this study, we developed a predictive model to identify the patients most likely to benefit from surgery. After adjusting for the baseline characteristics, the patients who received surgery and were not re-matched were removed to avoid selection bias. Patients from the surgery group had significantly longer OS compared to patients in the non-surgery group (46 vs. 31 months). Based on the median OS data from the non-surgery group (31 months), the surgery patients were classified into beneficial and non-beneficial groups in which the patients who underwent local resection of the primary tumor had lived longer than the 31 months benefit from surgery. Next, the preoperative baseline characteristics were compared between the beneficial and non-beneficial groups. Multivariate logistic regression was used to identify the risk factors associated with the benefit from surgery. These data were used to construct a predictive nomogram to identify the optimal MBC patients for surgery.

In our model, BC subtype had the strongest impact on patient benefit from surgery. The HR^+^/HER2^+^ subtype had the best score, followed by the HR^−^/HER2^+^ and HR^+^/HER2^−^ subtypes. The worst score was observed in patients with the HR^−^/HER2^-^ subtype. A prospective trial in Turkish patients suggested improvements in 5-year survival following surgery in patients with HR ( +) BC^28^. Also, a multicenter study in the Netherlands demonstrated that MBC patients with HR + /HER2 + disease had longer survival^29^. However, there was no notable treatment outcome in patients with triple-negative breast cancer^30,31^. These results indicate that for patients with advantaged molecular BC subtypes, surgery is a beneficial therapeutic option.

Liver metastasis was also shown to strongly predict patients who may not benefit from surgery. Compared to metastasis to the bones, lung or brain, BC with liver metastasis is regarded as the most lethal form of the disease which has a 5-year survival rate of 3.8–12%^32^. Walsum et al.^33^ found that treatment options for BC patients with liver metastasis generally remain restricted to palliative systemic therapy. Given that it is relatively uncommon for isolated liver metastasis to occur in BC, local treatment is still rarely proposed and so the usual treatment modalities may not be suitable for BC patients with liver metastasis. Primary tumor resection may play a minor role in prolonging the survival of patients with MBC.

In our study, marital status and younger age were found to be associated with survival benefits after primary tumor resection. A recent study reported that surgery to the primary site can improve outcomes for younger MBC patients^28^. This may be because younger patients usually have a higher performance and physical status which helps them to tolerate surgery. Marital status is an independent prognostic factor for OS in MBC patients^24^. Considering the decreased psychological burden and increased emotional support of married patients, they may be more likely to benefit from primary tumor resection.

Wang et al.^34^ divided 2056 patients with breast infiltrating duct carcinoma (BIDC) into surgery and non-surgery groups according to whether or not surgery was undertaken, and showed that resection of the primary tumor was associated with improved survival in MBC patients. At the same time, they constructed a nomogram based on the independent factors affecting the benefit of locoregional surgery in patients with stage IV BIDC to predict the probability of surgical benefit. In their model^34^, stage IV BIDC patients with low histologic grade, low T-stage, non-triple-negative subtype, non-lung metastasis, non-liver metastasis, non-brain metastasis, or married status were regarded as more suitable candidates for resection. On the basis of these previous studies, we extended the inclusion criteria to all pathological types of stage IV BC patients, not just patients with stage IV BIDC, allowing greater coverage by the model. In addition, it is worth noting that we also used the Chinese population as an external validation of the model, and the results showed that the model still had good predictive ability. But this study had several potential limitations. The study was retrospective and so may be impacted by selection bias related to the study design. Also, specific information about systemic treatments such as HER2 targeted therapy, endocrine therapy and immunotherapy were lacking in the SEER database and so it is unclear if primary tumor resection combined with therapies could result in survival benefits. Finally, important details including the number of metastatic sites, complications and treatment duration were unavailable in the SEER database. Further validation of our data is required in large-scale prospective studies.

In summary, our predictive model showed that surgery to the primary tumor had a beneficial effect on the OS of patients with MBC yet not all were amenable to surgery. Specifically, patients < 60 years old, those with grade I tumors with diameters < 20 mm, non-liver metastasis, HR + /HER2 + and married patients who are eligible for primary tumor resection and could potentially benefit more from surgery. Our predictive model should be considered for use in clinical practice as it has the potential to identify the optimal candidates for surgery to the primary site in MBC patients.

## Data Availability

The datasets used during the current study are available from the corresponding author on reasonable request.
